# Elucidation of how the *Mir-23-27-24* cluster regulates development and aging

**DOI:** 10.1038/s12276-024-01266-3

**Published:** 2024-06-14

**Authors:** Xin Le Yap, Jun-An Chen

**Affiliations:** 1 Molecular and Cell Biology, Taiwan International Graduate Program, Academia Sinica and Graduate Institute of Life Sciences, National Defense Medical Center, Taipei, Taiwan; 2 Institute of Molecular Biology, Academia Sinica, Taipei, Taiwan; 3 Neuroscience Program of Academia Sinica, Academia Sinica, Taipei, Taiwan

**Keywords:** miRNAs, Ageing

## Abstract

MicroRNAs (miRNAs) are pivotal regulators of gene expression and are involved in biological processes spanning from early developmental stages to the intricate process of aging. Extensive research has underscored the fundamental role of miRNAs in orchestrating eukaryotic development, with disruptions in miRNA biogenesis resulting in early lethality. Moreover, perturbations in miRNA function have been implicated in the aging process, particularly in model organisms such as nematodes and flies. miRNAs tend to be clustered in vertebrate genomes, finely modulating an array of biological pathways through clustering within a single transcript. Although extensive research of their developmental roles has been conducted, the potential implications of miRNA clusters in regulating aging remain largely unclear. In this review, we use the *Mir-23-27-24* cluster as a paradigm, shedding light on the nuanced physiological functions of miRNA clusters during embryonic development and exploring their potential involvement in the aging process. Moreover, we advocate further research into the intricate interplay among miRNA clusters, particularly the *Mir-23-27-24* cluster, in shaping the regulatory landscape of aging.

## miRNA clusters: key regulators of development and aging dynamics

MicroRNAs significantly influence everything from growth to aging. However, the specific roles of certain miRNA clusters, like *Mir-23-27-24*, in aging are less understood. This review investigates the *Mir-23-27-24* cluster’s function in embryonic growth and possible aging regulation. It’s a comprehensive review of how the *Mir-23-27-24* cluster affects vital biological processes and aging, combining existing research to fill knowledge gaps. The *Mir-23-27-24* cluster includes miRNAs vital in development stages and linked to various diseases, including cancer. Researchers underline the cluster’s role in embryonic development and its overlooked role in aging. They explore how these miRNAs control gene expression and contribute to the complexity of biological functions. This study implies that miRNA clusters like *Mir-23-27-24* could be crucial in aging, opening new paths for aging research and potential treatments for age-related diseases.

This summary was initially drafted using artificial intelligence, then revised and fact-checked by the author.

## Introduction

MicroRNAs (miRNAs) are short noncoding RNAs known to be involved in gene regulation, and they play unique roles in various organismal systems and processes, spanning early development through aging^1–3^. An extensive body of literature has shown that miRNAs play important roles in the development of eukaryotes, as deletion of essential miRNA biogenesis-related proteins induces early lethality^1^. In addition, miRNAs play critical roles in regulating diverse biological pathways, and their dysregulation can lead to premature aging, particularly in nematodes and flies^4^. Optimal miRNA expression is crucial for maintaining homeostasis, and miRNA selectivity enables the spatial and temporal specificities of miRNAs since miRNA expression differs among tissues and developmental stages^5^. miRNAs that are especially important in critical biological pathways are highly conserved across species, with some occurring as clusters to enable more efficient execution of their functions. miRNA clusters are defined as those that overlap within a primary transcript, are located in adjacent genome loci and are transcribed in the same direction^6^. Generally, miRNA clusters comprise two to three miRNAs, yet large miRNA clusters also occur, such as the chromosome 19 miRNA cluster (C19MC), which is the largest in the human genome, comprising 46 miRNAs that span ~100 kilobases^7^. Since individual miRNAs have various target genes, a miRNA cluster ultimately evolves complex regulatory activity, thereby influencing a diverse array of biological processes. Among these miRNA clusters, the *Mir-17-92* cluster and *Mir-23-27-24* cluster are two of the best characterized and reflect critical functions in embryonic development and disease. As the function of the *Mir-17-92* cluster has been extensively reviewed previously^8,9^, in this review, we use the *Mir-23-27-24* cluster as an example to elaborate on why miRNAs tend to cluster together. Although the roles of the *Mir-23-27-24* cluster in various diseases, such as cancer, have been relatively well documented^10–12^, its functions in normal physiological processes from embryonic development to tissue homeostasis during aging are less well understood. Specifically, its involvement in regulating aging is underappreciated, with only sparse and inconclusive experimental evidence supporting its role in this context. Thus, we provide a summary of the role of the *Mir-23-27-24* cluster in development, followed by a discussion linking these functions to its regulation of the aging process. Hereafter, for clarity in this review, we follow the recommendations of the most updated miRNA annotation consortium, using “*Mir-23-27-24*” and “MiR23, MiR27, and MiR24” to specifically indicate the mouse gene and mature forms of the miRNAs, respectively, and annotate the respective human forms as “*MIR-23-27-24*” and “MIR23, MIR27, and MIR24”^13^. In the following sections, we summarize the roles of the *Mir-23-27-24* cluster in development and link its activity to aging regulation.

**Fig. 1:**
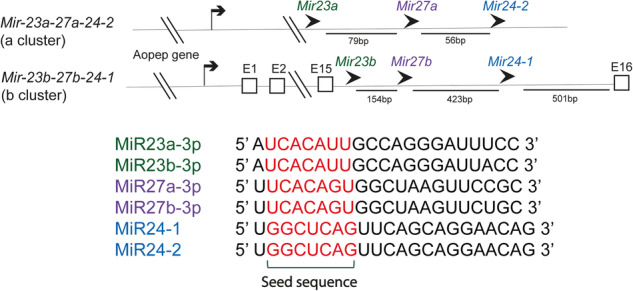
The *Mir-23-27-24* cluster. Sequence alignment of the mouse *Mir-23-27-24* cluster with the corresponding seed sequences highlighted in red.

In most vertebrates, the *Mir-23-27-24* cluster comprises two paralogs resulting from a gene duplication event: Cluster a (*Mir-23a-27a-24-2*) and Cluster b (*Mir-23b-27b-24-1*) (Fig. 1)^14^. *Mir-23a-27a-24-2* is located intergenically, whereas *Mir-23b-27b-24-1* is located intronically within the aminopeptidase O (*Aopep*) gene. The MiR23, MiR27 and MiR24 miRNA gene families are involved in both gene clusters, with a total of five miRNAs being produced from these miRNA loci (MiR24-1 and MiR24-2 possess an identical mature MiR24 sequence). Although the two paralogous clusters are located on different chromosomes, both harbor three similar miRNAs, with each pair having the exact same seed sequence. Although miRNAs can target the same set of mRNAs based solely on their identical seed sequences, their expression and roles vary across tissues, likely due to versatile transcription and post-transcriptional regulation^15^. Although these clusters are highly conserved across vertebrates, one cluster is not found in chickens or rabbits. Thus, even though these two paralogous clusters share the same seed sequences, their functions may not be completely identical. The dynamic spatiotemporal expression pattern of the *Mir-23-27-24a*/*b* clusters likely increases the complexity of their functions during embryonic development, as many of their target genes might be tightly regulated in the same biological pathways^14^. This concept is aptly demonstrated by the *Mir-17-92* cluster, in which each member may exhibit either cohesive or divergent functions. The *Mir-17-92* cluster comprises MiR17, MiR18, MiR19a/b, MiR20, and MiR92. Notably, although the *Mir-17-92* cluster functions as a potent oncomir in cancer progression, MiR19a/b has emerged as the primary cluster member responsible for recapitulating the entire cluster phenotype^16,17^. Conversely, during spinal motor neuron generation, the *Mir-17-92* cluster exhibits a cohesive mode of action. In this scenario, MiR19a/b targets phosphatase and tensin homolog (PTEN), whereas MiR17/20 targets the E3 ubiquitin ligases Nedd4 family interacting protein 1 (Ndfip1) and neural precursor cell expressed developmentally downregulated 4-like (Nedd4-2) to regulate PTEN monoubiquitination^18,19^. Accordingly, knocking out the *Mir-17-92* cluster in motor neurons leads to concomitant increases in the expression of PTEN and Ndfip1/Nedd4–2, thereby promoting the translocation of monoubiquitinated PTEN to the nucleus, where it induces motor neuron apoptosis. Thus, individual miRNAs within the *Mir-17-92* cluster can coherently regulate the subcellular localization of their target proteins. This scenario might provide an explanation for why miRNAs are generally clustered in mammalian genomes, as clustering allows them to orchestrate target functions by coordinately regulating gene expression and post-translational modifications^18,19^.

## Role of the *Mir-23-27-24* clusters in development

Many miRNA clusters have been recognized as critical regulators during development, as germline deletion of some miRNA clusters tends to be embryonically lethal or results in organ defects^20^. The *MIR-17-92* cluster is important for normal development, and it was also the first miRNA cluster to be implicated in human diseases, such as Feingold syndrome^21^. This cluster is often dysregulated in hematopoietic and solid cancers. Germline deletion of the *Mir-17-92* cluster is embryonically lethal, resulting in prominent septal defects and impaired B-cell development^20^. Moreover, knockout of the *Mir-17-92* cluster in a tissue-specific manner also causes notable defects. For example, the deletion of *Mir-17-92* in spinal motor neurons induces partial perinatal lethality and gross locomotive deficits^18,19^.

### Stem cell differentiation

**Fig. 2:**
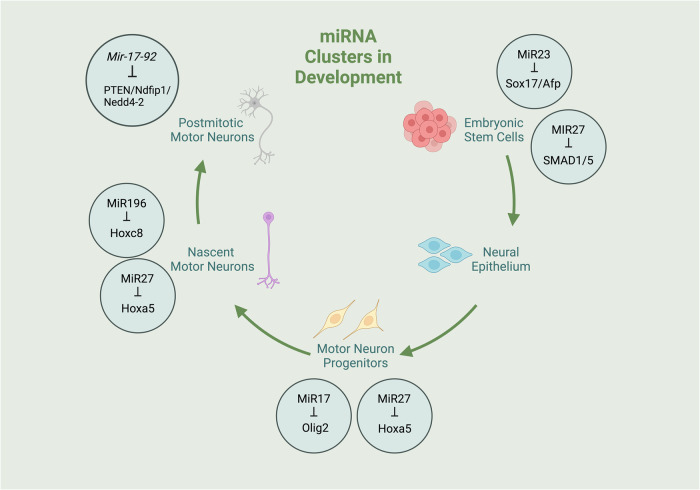
miRNA clusters involved in development. *Mir-23-27-24* and *Mir-17-92* play important roles throughout development. The figures were created with BioRender.com.

Another pivotal miRNA cluster for mammalian embryonic development is *Mir-23-27-24* (Fig. 2). At the early stage of development, the paralogous *MIR-23-27-24* clusters are highly expressed during stem cell differentiation. For example, during hepatocyte differentiation, both MIR23b and MIR27b are upregulated during the transition of human embryonic stem cells (hESCs) into hESC-derived definitive endoderm cells^22^. Similarly, MIR24 is upregulated during the transition of hESC-derived definitive endoderm cells into hepatocytes^22^. Furthermore, MIR23a, MIR23b, MIR27a and MIR27b were found to be enriched in the definitive endoderm stage, as was MIR23 in the hepatocyte stage^22^, demonstrating the importance of these miRNAs in closely regulating the different stages of stem cell differentiation. MiR27a and MiR24 also act as suppressors of ESC self-renewal, and inhibiting the expression of these molecules promotes somatic cell reprogramming^23^. Knockout analysis involving loss of both MiR27 and MiR24 revealed somatic cell reprogramming and severe defects in mesoderm differentiation of ESCs^23^. Notably, the same study revealed that MIR23a-3p is one of the few miRNAs enriched in mouse embryonic fibroblasts, and this molecule is also upregulated during embryoid body differentiation. This study further established the role of the *Mir-23-27-24* cluster in early development and demonstrated that its members exert their own specific functions.

In contrast to MiR27a and MiR24, MiR23a represses endoderm and ectoderm differentiation by suppressing two differentiation markers, SRY-box transcription factor 17 (Sox17) and alpha fetoprotein (Afp)^24^. A notable increase in the activation of mouse endodermal genes, including *Afp*, *Sox17*, and GATA binding protein 6 and 4, and ectodermal genes, such as ISL LIM homeobox 1 (*Islet1*), fibroblast growth factor 5 (*Fgf5*), and SRY-box transcription factor 1 (*Sox1*), was observed following MiR23a inhibition, although markers for trophectoderm and mesoderm lineages remained unaffected. Notably, MiR23a overexpression in mouse ESCs suppressed differentiation toward these lineages, suggesting that MiR23a plays a crucial role in maintaining pluripotency. These findings unequivocally establish MiR23a as an additional regulator of ESC differentiation. In addition to the endoderm lineage, mesendoderm formation is also a crucial step in embryogenesis and is controlled by bone morphogenetic signaling. In this case, MIR27b has been found to play an important role by repressing mediators of bone morphogenetic protein signaling, e.g., phosphorylated SMAD1/5, throughout the definitive endoderm differentiation of human induced pluripotent stem cells by promoting mesoderm formation^25^.

### Noise buffering in neuronal progenitors

The *Mir-23-27-24* cluster is also involved in orchestrating tissue morphogenesis during embryonic development. For example, the *Mir-23-27-24* cluster plays multifaceted roles during neural development. Upon neural tube closure, the initial rostrocaudal patterning of the neural tube leads to differential expression of *Hox* genes that contribute to the specification of motor neuron subtype identity^26^. Although several *Hox* mRNAs are expressed in motor neuron progenitors in a fluctuating manner, the Hox proteins are not expressed in these progenitors and become detectable only in postmitotic motor neurons^27^. Interestingly, the homeobox A5 (Hoxa5) protein is precociously expressed in progenitors, and the Hox5/8 boundary is expanded caudally in conditional mutants for *Dicer* (encoding a principle enzyme for miRNA biogenesis), both in vitro and in vivo. In silico simulations revealed that two feed-forward Hox-miRNA loops account for precocious and fluctuating Hoxa5 expression, as well as the ill-defined boundary phenotype of *Dicer* mutants^27,28^. Within the *Mir-23-27-24* cluster, MiR27 appears to be a major regulator coordinating the temporal delay and spatial boundary of Hox protein expression^27^. These results describe a novel Hox-miRNA circuit that filters transcription noise and controls the timing of protein expression to confer robust individual motor neuron identity. More interestingly, the same set of miRNAs participates in two feed-forward loops, with the potential to buffer transcriptional noise and sharpen the boundary. This novel miRNA-mediated mechanism represents a powerful strategy for endowing precision and robustness to morphogen-mediated pattern formation^27^.

### Establishment of neuronal identity via bistability

In addition to its impacts on neural progenitors, MiR27 also participates in tuning the establishment of neuronal identity^29^. The lineage commitment of spinal motor neurons in the cervical region is marked by a clear segregation of two lineage-determining transcription factors, e.g., Hoxa5 and Hoxc8^30^. However, using single-cell RNA sequencing and mouse genetic lineage tracing, our group revealed that the fate decisions of the cells at the boundary are achieved without segregating the mRNAs of the two *Hox* genes^29^. Furthermore, transcriptional cross-repression between these two factors, i.e., through a classical feedback loop, does not occur. These observations challenge existing paradigms of the feedback mechanisms underlying cell fate decisions. This novel type of feedback mechanism has not been reported previously, and it is estimated that there are at least 10^4^ distinct mRNA‒miRNA reaction systems that match this bistability-enabling topology in human cells^29^. This new theory of miRNA‒mRNA circuits can be applied to a wide range of systems^31^ in addition to the already known rich dynamics of post-transcriptional reaction networks widespread in biology, along with a novel mechanism involved in regenerating multimodal gene expression in cell populations^32^.

## Roles of the *Mir-23-27-24* cluster in aging

**Fig. 3:**
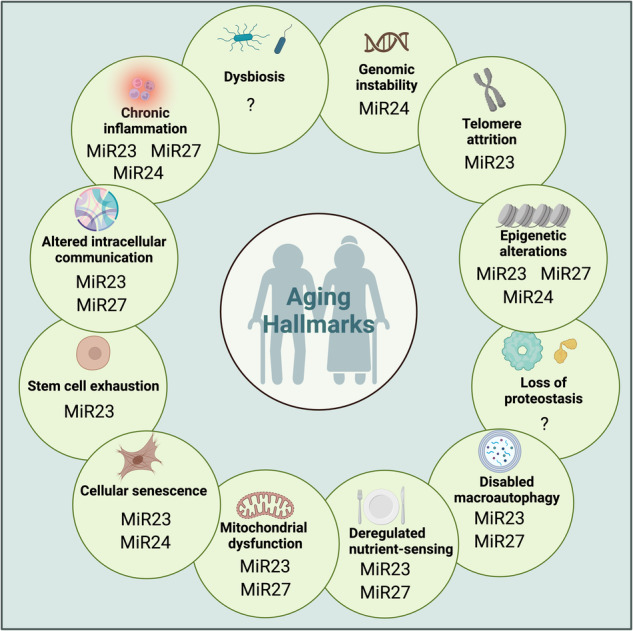
The *Mir-23-27-24* cluster during aging. Members of the *Mir-23-27-24* cluster contribute to the 12 hallmarks of aging. The figures were created with BioRender.com.

Aging is one of the most pressing socioeconomic and health problems of the twenty-first century. It represents the predominant risk factor for most human pathologies, including cardiovascular diseases, neurological disorders, and cancer. Medical interventions to impede the aging process would substantially impact human disease. Currently, aging is characterized by the progressive dysfunction of multiple organs and tissues within an organism. For almost all late-onset neurodegenerative disorders—such as Alzheimer’s disease, Parkinson’s disease, and amyotrophic lateral sclerosis—aging is the only known common risk factor^33^. Deciphering the molecular mechanisms underlying aging could not only reduce the prodigious costs of medical care for patients suffering from aging-induced pathologies and disorders but also provide a key step toward the long-held aspiration of human beings, e.g., the fountain of youth and healthy longevity. Not surprisingly, miRNAs have been identified as functional regulators of the aging process via a specific “regulome”. Not only do miRNAs manifest spatiotemporal expression patterns during embryonic development, but many studies have revealed distinctive patterns of miRNA expression in different organs upon aging^34,35^. However, more thorough research on this topic is warranted. Generally, aging can be characterized by several interconnected molecular hallmarks, including genomic instability, telomere attrition, epigenetic alterations, loss of proteostasis, disabled macroautophagy, deregulated nutrient-sensing, mitochondrial dysfunction, cellular senescence, stem cell exhaustion, altered intercellular communication, chronic inflammation, and dysbiosis (Fig. 3)^36,37^. Intriguingly, the *Mir-23-27-24* clusters participate in a series of molecular pathways contributing to these aging hallmarks, suggesting that they play underappreciated roles in the aging process. Notably, previous studies have reported conflicting results regarding the effects of overexpressing or deleting members of the *Mir-23-27-24* cluster on aging. These discrepancies can be attributed to the dual targeting modes of miRNAs, e.g., binary switches or tuning interactions^38^. In tuning interactions, optimal miRNA expression levels function as a rheostat, finely regulating target expression within physiological ranges. Deviations from this balance can lead to either excessive or insufficient target expression, impairing the ability of a cell or organism to respond effectively to stress. Moreover, MiR27 may undergo target-directed miRNA degradation (TDMD), complicating predictions about its overexpression effects^29,39^. Given that aging involves a complex interplay of various factors across different cell types and tissues, it is crucial to determine the physiological expression levels of each *Mir-23-27-24* cluster member and its targets in a cell-type-specific manner during aging. This knowledge will inform decisions about appropriate stoichiometry and expression levels when considering interventions to augment *Mir-23-27-24* cluster members to manipulate the aging process. Below, we explore the roles of the *Mir-23-27-24* clusters in regulating various hallmarks of aging^37^, exemplifying how such miRNAs might contribute to aging regulation, whether miRNA clusters are more likely to be involved in manipulating the pace of aging (Fig. 3) and summarized the involved aging hallmarks in Table 1.

**Table 1:** *Mir-23-27-24* cluster-associated miRNAs and their links to the hallmarks of aging.

No	miRNA	Target	Aging hallmark	Cells or tissues examined	Ref.
1	MIR24	H2AX	Genomic instability	CD8 T	^48^
2	MIR24	BCL2L11 (BIM)		BEAS-2B	^49^
3	MIR24	p38, p53, PML and H2AX		HCT 116 and MCF-7	^47^
4	MIR-23a	TRF2	Telomere attrition	HEK293T	^54^
5	*MIR*-23-27-24	HIC1	Epigenetic dysregulation	MCF-7	^10^
6	MIR24-2	PRMT7		Human liver cancer stem cells (hLCSCs)	^63^
7	MiR23a	ASK-1	Disabled macroautophagy	Yak cumulus cells	^67^
8	MIR27	PINK1		HeLa and M17 cells	^68^
9	MIR23, MIR27	SPROUTY2	Deregulated nutrient-sensing	Human umbilical vein endothelial cells (hUVECs)	^72^
10	MiR23	Eif4ebp2		Mouse primary bone marrow-derived macrophages (BMDMs)	^73^
11	MIR27	MFF	Mitochondrial dysfunction	Human CHANG liver cells	^79^
12	MIR23a, MIR23b	GLS		Human P-493 B lymphoma cells, PC3 prostate cancer cells, CB33 lymphoblastoid cells, CB33-Myc cells and MCF7 human breast cancer cells	^80^
13	MIR24	TOP1	Cellular senescence	Human diploid fibroblasts	^84^
14	MIR23	HAS2		Dermal fibroblasts	^85^
15	MiR23	SMAD5	Stem cell exhaustion	Mouse embryonic stem cells	^90^
16	MiR23	Tmem64		Bone marrow mesenchymal stem cells	^91^
17	Mir27b	Pax3		Mouse primary satellite cells	^92^
18	MiR23	Fas	Altered intercellular communication	Mouse radiation-induced thymic lymphoma tissues	^95^
19	MIR27b	SMAD4		AML-12	^96^
20	MIR27a	SFRP1		HCT-116 and Caco-2 cell lines	^97^
21	MIR23b	TAB2, TAB3 and IKK-α	Chronic inflammation	Human arthritic joints	^100^
22	MIR23a	IKKα		Human articular chondrocytes	^101^
23	MiR23a	A20		Mouse BMDMs	^102^
24	MiR27a	IRF4, PPAR-γ			^102^
25	MiR24	Chi3l1		Mouse aortic aneurysm	^103^

### Genomic instability

Genome stability is crucial to the growth, development, functioning, and reproduction of all living organisms. In general, genomic instability arises from alterations in the structure or number of chromosomes, point mutations within genes, and other forms of genetic changes^40,41^. Although such instability can be a natural element of cellular processes, uncontrolled genomic instability results in diseases such as cancer and aging^42,43^. Dysregulation of miRNAs has been found to be a clear modulator of genomic instability, with emerging research centering on cancer and the DNA damage response^44–46^.

The *MIR-23-27-24* clusters potentially play a role in human aging by regulating several important DNA damage response genes^37,38^, such as H2A.X variant histone (*H2AX)* and BCL2 like 11 (*BCL2L11*), as well as melatonin-activated pathway genes, such as mitogen-activated protein kinase 14 (*MAPK14*), tumor protein p53 (*TP53*), and PML nuclear body scaffold (*PML*)^47^. This role is exemplified in post-thymic CD8+ T cell differentiation when the *MIR-23-27-24* cluster is upregulated^48^. In that study, increased MIR24 expression reduced the expression of the histone variant H2AX, which is crucial for DNA damage response pathways. Melatonin can reduce DNA fragmentation only when MIR24 is expressed at physiological levels, indicating that the ability of melatonin to protect cells from DNA damage requires the downregulation of MIR24 expression^47^. Another study reported the ability of MiR24 to inhibit BRCA1 in the homologous recombination DNA repair pathway^49^, with BRCA1 expression inversely correlated with MiR24 levels in lung tissue specimens from a chronic obstructive pulmonary disease model.

### Telomere attrition

Telomeres are specialized regions of repetitive DNA sequences at the termini of chromosomes that become shorter with each cell division, indicating that telomere length is a good marker in the context of aging and age-related diseases such as AD and chronic obstructive pulmonary disease^50^. Shelterin, or telosome, a protein complex associated with telomeres, ensures telomere integrity, safeguards telomeres from being recognized as DNA breaks, and synchronizes telomerase-dependent maintenance of telomere length^51^. Many miRNAs have been linked to regulating proteins in the shelterin complex. For instance, elevated MIR185 promotes telomere elongation and simultaneously accelerates the replicative senescence process in a protection of telomeres 1 (POT1)-dependent manner^52^. miRNAs also contribute to mechanisms to maintain telomere length, such as telomerase activity and alternative lengthening of telomeres (ALT). For example, MIR708 was shown to be highly expressed in a large panel of cells that underwent ALT, and its overexpression suppressed cell migration, invasion, and angiogenesis^53^.

Through its targeting of telomeric repeat binding factor 2 (TRF2), a double-stranded DNA binding protein important for protecting telomere ends and T-loop formation, MIR23a of the *MIR-23-27-24* clusters has been associated with telomere dysfunction. Overexpressing MIR23a in human primary fibroblasts ultimately limited TRF2 targeting to telomere chromatin, leading to telomere dysfunction-induced foci and ataxia-telangiectasia-associated signaling activation^54^. Notably, the same group reported increased senescence in MIR23a-overexpressing cells, and this effect was reversed upon coexpression of exogenous TRF2, indicating that MIR23a regulates telomere maintenance and senescence by directly inhibiting TRF2^55^.

### Epigenetic dysregulation

Various epigenetic alterations contribute to the aging process, including alterations in the acetylation and methylation of DNA or histones and in the levels or activity of chromatin-associated proteins or noncoding RNAs (ncRNAs)^56^. Numerous reviews have summarized and demonstrated a strong link between miRNAs and epigenetic regulation, with other ncRNAs also participating in the epigenetic landscape^57,58^. miRNAs can function as epigenetic modulators by acting on enzymes important in epigenetic reactions, such as DNA methyltransferases and histone methyltransferases. Concurrently, the epigenetic landscape regulates miRNAs by governing DNA methylation, RNA modification and histone modification on these small ncRNAs^59–61^. For example, DNA methylation of the promoter region of MIR410 is more prominent in glioma tissues, resulting in reduced MIR410 expression in gliomas, and gain- and loss-of-function experiments further support that MIR410 significantly controls cell growth, cell cycle progression, and glioma cell apoptosis^62^. Such epigenetic mechanisms regulate gene expression without affecting genome sequences.

The *MIR-23-27-24* cluster has been shown to target hypermethylated in cancer 1 (*HIC1*). Interestingly, HIC1 represses transcription to control the expression of the *MIR-23-27-24* clusters by binding to HIC1-binding motifs, forming a double-negative feedback loop that ultimately contributes to breast cancer progression^10^. MIR24-2 has also been reported to be involved in many aspects of epigenetic regulation by targeting protein arginine methyltransferase 7 (PRMT7), thereby inhibiting dimethylation/trimethylation of histone H4 arginine 3 and eventually promoting the expression of Nanog via hepatocellular carcinoma-upregulated long noncoding RNA (HULC)^63^. Another study revealed the importance of MIR24-2 in epigenetic regulation since it increases the expression of both the N6-adenosine-methyltransferases METTL3 and MIR6079 via RNA methylation. Lysine demethylase 4A (JMJD2A), a target of MIR6079, promotes the trimethylation of histone H3 on the ninth lysine (H3K9me3)^64^. Moreover, MIR24-2 impacts several epigenetics-related genes, including *pHistone H3*, SUZ12 polycomb repressive complex 2 subunit (*SUZ12*), histone lysine methyltransferase *SUV39H1*, *Nanog*, mitogen-activated protein kinase kinase kinase 4 (*MEKK4*) and phosphotyrosine (*pTyr*)^64^. Together, these findings demonstrate that MIR24-2 of the *MIR-23-27-24* cluster is an important epigenetic regulator and thus might be crucial in the aging process.

### Disabled macroautophagy

Autophagy is a cellular process crucial to cell survival that involves the degradation and recycling of cellular components, such as damaged organelles and proteins, to maintain cellular health and homeostasis. This intracellular catabolic process encompasses microautophagy, macroautophagy and chaperone-mediated autophagy. In macroautophagy, engulfed substrates are sequestered within cytosolic double-membrane vesicles, termed autophagosomes^65^. Macroautophagy targets nonproteinaceous macromolecules and entire organelles, and when this process is impaired, such as during aging, organelle turnover and responsiveness to environmental stresses can be negatively impacted. Notably, extracellular vesicle-encapsulated *mir*-*83* in *Caenorhabditis elegans* represses the expression of an autophagy regulator, Mucolipin 1 (CUP-5/MCOLN), with *mir*-*83* expression levels increasing with age in the intestine, highlighting a role for this miRNA in coordinating macroautophagic events during the aging process^66^.

Similarly, MiR23a has been strongly linked to autophagy because it targets apoptosis signal-regulating kinase 1 (ASK-1), limiting the apoptosis of cumulus cells in yaks (*Bos grunniens*)^67^. Moreover, both MIR27a and MIR27b regulate mitochondrial autophagy by repressing the mRNA of PTEN-induced putative kinase 1 (PINK1)^68^, thereby preventing the PINK1 accumulation that occurs upon mitochondrial damage and curtailing mitophagic influx. Interestingly, under chronic mitophagic flux, the expression of both of these miRNAs was significantly upregulated, indicating a negative feedback mechanism between PINK1-mediated mitophagy and the *MIR-23-27-24* cluster.

### Deregulated nutrient-sensing

Diet has a powerful modulatory influence on aging, and caloric restriction has emerged as a valuable intervention in this regard^69^. However, many questions about how caloric intake controls aging-related processes remain unanswered. Nutrient-sensing pathways become dysregulated and lose effectiveness with age. Fully understanding the underlying mechanisms is a critical step for discovering therapeutic strategies. Diet has also been shown to have an important influence on miRNAs. Notably, certain miRNAs affect proteins and enzymes involved in nutrient-sensing pathways and thus may contribute to modulating the aging process. For instance, reduced levels of the miRNA lin-4 in *C. elegans* decreased the lifespan and accelerated tissue aging, whereas overexpressing lin-4 extended life through the insulin/insulin-like growth factor-1 pathway^70^. Moreover, the p53 homolog in *Drosophila* (Dp53) is synchronized by *miR*-*305* in a nutrient-dependent manner. In well-fed flies, Target of Rapamycin (TOR) signaling results in *miR*-*305*-mediated inhibition of Dp53, whereas nutrient deprivation reduces the levels of *miR*-*305* and promotes Dp53 derepression, indicating a role for Dp53 targeting by *miR*-*305* in nutrient-sensing and metabolic adaptation^71^.

The involvement of the *Mir-23-27-24* cluster in sensing and regulating extrinsic signals related to aging is also noteworthy. Both MiR23 and MiR27 have been implicated in deregulated nutrient-sensing through their suppression of Sprouty2 (SPRY2) and Semaphorin 6A (Sema6A), respectively, which negatively regulate Ras/mitogen-activated protein kinase (Ras/MAPK) and vascular endothelial growth factor receptor 2 (Vegfr2)-mediated signaling, thereby promoting angiogenesis^72^. Moreover, both the MAP and phosphatidylinositol 3’-kinase (PI3K)-Akt kinase signaling pathways (which are also important in nutrient-sensing) are activated when vascular endothelial growth factor (Vegf) binds to its receptors. In contrast, loss of MiR23/27 impaired MAPK and Vegfr2 signaling in response to Vegf, resulting in angiogenic suppression. Loss of the *Mir*-*23-27-24* cluster in the myeloid lineage of mice elicits an interesting phenotype, in which these mice gain less weight on a high-fat diet than controls while simultaneously exhibiting exacerbated glucose and insulin tolerance owing to a reduced population of lipid-associated macrophages^73^. The authors of that study attributed this outcome to MiR23 targeting eukaryotic translation initiation factor 4E binding protein 2 (*Eif4ebp2*), an essential gene that restricts protein synthesis and proliferation in macrophages. Thus, the *Mir-23-27-24* clusters can impact different hallmarks of aging, including deregulated nutrient-sensing, loss of proteostasis, and chronic inflammation.

### Mitochondrial dysfunction

As cell powerhouses, mitochondria contribute significantly to the aging phenotype. Mitochondrial function starts to deteriorate upon aging owing to multiple intertwined mechanisms involving other aging hallmarks, including the accumulation of mitochondrial DNA mutations, destabilization of respiratory chain complexes, reduced mitochondrial turnover, and changes in mitochondrial dynamics. These events tend to increase the production of reactive oxygen species (ROS), which alter mitochondrial membrane permeability and promote inflammation and autophagy^74^. A group of miRNAs located in or associated with mitochondrial functions, collectively termed mitomiRs, trigger macrophage differentiation and modulate their downstream activation and immune functions^75^. Moreover, mitomiRs contribute to cardiac diseases and have proven important in elucidating how miRNAs regulate mitochondrial RNAs in cells lacking mitochondrial DNA^76,77^. For example, during muscle differentiation, the myogenesis-specific miRNA MIR1 enters mitochondria to activate the translation of various mitochondrial genome-encoded transcripts^78^.

In the *MIR-23-27-24* cluster, MIR27 targets the 3′ untranslated region (UTR) of the mRNA of mitochondrial fission factor (MFF) to limit its expression. In doing so, MIR27 regulates mitochondrial dynamics by controlling mitochondrial elongation, membrane potential, and ATP levels^79^. Furthermore, both MIR23a and MIR23b are downregulated by c-Myc, an oncogenic transcription factor, resulting in impaired mitochondrial glutaminase (GLS) activity and reactive oxygen species homeostasis, thereby increasing glutamine catabolism to promote ATP production and glutathione synthesis^80^.

### Cellular senescence

Cells undergo cellular senescence primarily due to chronic stress or cellular damage, a process that is triggered at least in part by telomere shortening with aging. Given the considerable stress on aging cells, many cells enter a cellular senescence state to suppress replication, ultimately perturbing organismal homeostasis. A set of miRNAs—including MIR210, MIR376a*, MIR486-5p, MIR494, and MIR542-5p—induce double-strand DNA breaks and ROS accumulation as a consequence of cell senescence^81^. The miRNA let-7 interacts with argonaute RISC catalytic component 2 (AGO2), which accumulates in senescent cells and silences gene transcription^82^. Thus, miRNAs are involved in senescence either by inducing senescence through double-strand DNA break accumulation or gene silencing.

The paralogous *MIR-23-27-24* clusters also regulate this aging hallmark, with MIR24 overexpression inducing the downregulation of DNA topoisomerase I (TOP1), an enzyme responsible for controlling and altering the topological state of DNA during transcription, which is especially important for maintaining genome stability and organization^83^. MIR24-induced downregulation of TOP1 causes DNA damage and stabilizes p53, providing favorable conditions for fibroblasts to undergo stress-induced premature senescence^84^. Moreover, MIR23a has been linked to senescence-associated skin aging by targeting the polysaccharide hyaluronan synthase 2 (HAS2) in the extracellular matrix^85^. Overexpression of MiR23a in nonsenescent mouse fibroblasts reduces Has2 levels and increases those of senescence-associated markers in mice, mimicking the normal aging process in vivo.

### Stem cell exhaustion

Stem cell differentiation is critically important since it enables differentiated cell types to perform their specific functions, especially during development. During aging, this process facilitates tissue repair upon injury or pathogenic infection. Certain miRNAs are important regulators of stem cells, and the loss of the primary enzymes responsible for miRNA biogenesis, such as Dicer or the microprocessor complex subunit DGCR8, impairs ESC differentiation^86^. For example, MiR145, MiR296, MiR470 and MiR134 target transcription factors such as POU class 5 homeobox 1 (Oct4) and SRY-box transcription factor 2 (Sox2) to regulate stem cell differentiation^87,88^. Notably, both ESCs and oocytes lack an antiviral mechanism because they are intrinsically incapable of producing interferons, a feature attributable to MiR673-driven control of mitochondrial antiviral signaling protein (MAVS), which regulates interferon production^89^.

In ESCs, the *Mir-23-27-24* cluster is regulated by bone morphogenetic protein 4 (Bmp4), which recruits phosphorylated SMADs to the promoter of the gene encoding the miRNA clusters. Instead of affecting self-renewal or pluripotency, this regulatory mechanism promotes ESC differentiation, inducing apoptosis in epiblast stem cells upon their differentiation. MiR23 also targets SMAD family member 5 (Smad5), a transcription factor downstream of the Bmp4 receptor, increasing the complexity of this *Mir-23-27-24*/apoptosis regulatory loop^90^. Furthermore, both MiR23a and MiR23b play a role in the differentiation of bone marrow mesenchymal stem cells into osteoblasts by targeting transmembrane protein 64 (Tmem64)^91^. Notably, excess expression of MiR23a and MiR23b promotes osteogenic differentiation, whereas inhibiting these miRNAs induces adipogenic differentiation, revealing a potential therapeutic approach for age-related osteoporosis. Although the role of the *Mir-23-27-24* clusters in the stem cell differentiation process is clear, their function in the stem cell exhaustion process during aging is less clear. One example is the targeting of the 3′ UTR of paired box 3 (*Pax3*) mRNA by MiR27b, which is required for the maintenance of skeletal muscle stem cells and their migration, both in the embryonic and adult stages^92^. By altering the expression of MiR27b, stem cell proliferation and the onset of differentiation are greatly affected, indicating that the regulation of Pax3 by MiR27b is crucial for the myogenic differentiation program in skeletal muscle.

### Altered intercellular communication

Communication between cells is essential for normal homeostatic functions, and cells displaying compromised communication ultimately exhibit deterioration of tissue health. The reduction in or loss of communication that arises during aging results in increased system noise encompassing both homeostatic and hormetic regulation, which is relevant to whether aging occurs systemically or begins from a certain tissue. miRNAs play an important role in intercellular communication, as they can be transported in extracellular vesicles within various body fluids—including plasma, serum, cerebrospinal fluid and urine—to regulate mRNA expression in remote target cells, including tumor and diseased cells^93^. miRNAs can also travel through gap junctions between cells in a connexin-dependent manner, thereby enabling coordinated cell proliferation and differentiation^94^. This scenario reveals another interesting facet of how miRNAs regulate the aging process, as they can be trafficked during multicellular development to specific organs, where they can synchronize both cell proliferation and differentiation.

The roles of the *Mir-23-27-24* cluster in altering intercellular communication have also been explored. Inhibiting both MiR23a and MiR23b in lymphoma cells increased the rate of cell death and apoptosis due to their binding to the cell surface death receptor (Fas) mRNA, which encodes a cellular signaling protein^95^. Intriguingly, that study revealed that MiR23a was much more effective at repressing Fas than was MiR23b, with the stronger regulatory effect of MiR23a attributable to an additional sequence beyond the conserved seed sequence, providing further evidence that despite having the same seed sequences, miRNAs can still exert tissue specificity. MiR27b has also been found to affect various signaling pathways by targeting SMAD family member 4 (Smad4), resulting in increased levels of Wnt family member 1 (Wnt1), β-catenin, c-Myc, and cyclin D1^96^. In contrast, inhibiting MIR27a expression upregulated secreted frizzled-related protein 1 (SFRP1), suppressed Wnt/β-catenin signaling, and significantly reduced cyclin D1 expression levels^97^.

### Chronic inflammation

One of the most important hallmarks of aging is chronic inflammation, reflected by elevated levels of proinflammatory markers in cells and tissues arising from dysregulation of immune cells, defective immunosurveillance or loss of self-tolerance. Many miRNAs are involved in these effects, either in their encapsulated form within extracellular vesicles for transport between different macrophages^98^ or by mediating inflammation-induced suppression of neural stem cell self-renewal, as is the case for MIR155^99^.

In the context of *MIR-23-27-24*, MIR23b is downregulated by interleukin 7A (IL-17), which contributes to rheumatoid arthritis. MIR23b can mitigate this effect by suppressing the expression of TGF-β-activated kinase 1/MAP3K7 binding protein 2 (TAB2), TAB3 and inhibitor of nuclear factor κ-B kinase subunit α (IKK-α), reflecting its potential as a target for inflammatory autoimmune therapeutic strategies^100^. MIR23a, which shares an identical seed sequence with MIR23b, also regulates inflammation by targeting IKKα in primary articular chondrocytes to inhibit IL-17-induced proinflammatory mediators^101^. Another research group revealed how the miRNAs of this cluster in macrophages activate the NF-ĸB proinflammatory pathway while inhibiting anti-inflammatory pathways^102^. In this case, MiR23a activates the NF-κB pathway by targeting TNF alpha-induced protein 3 (A20), thereby promoting proinflammatory cytokine production. MiR23a and MiR27a suppress Jak1/Stat6 and Irf4/Ppar-γ of the Jak1/Stat6 pathway, respectively, again supporting how the *Mir-23-27-24* clusters adopt a double-negative feedback loop in macrophage polarization networks, further providing evidence of their key regulatory activity in cancer progression. Another member of the cluster, MiR24, targets chitinase 3-like 1 (Chi3l1) to regulate cytokine synthesis and macrophage survival, and it also promotes aortic smooth muscle cell migration and cytokine production, leading to abdominal aortic aneurysms^103^, highlighting a novel plasma biomarker for monitoring the progression of this specific type of aneurysm. Together, these findings illustrate the role of these paralogous miRNA clusters in different biological contexts through divergent concentrations in different tissues despite having the same seed sequence.

## Future perspectives

In this review, we present *Mir-23-27-24* as an exemplary model to succinctly outline the implications of miRNA clusters in both developmental processes and aging. Although the importance of the *Mir-23-27-24* cluster in embryonic development is well established, its precise physiological roles in tissue maturation and aging remain unclear, primarily due to the prevailing focus of current research on specific pathways, resulting in a lack of studies of the broader systemic roles of such clusters. Future investigations could elucidate the nuanced involvement of *Mir-23-27-24* in aging. First, although the *Mir-23-27-24* cluster contributes to numerous molecular hallmarks of aging, existing studies often rely on in vitro cell culture models, providing only superficial insights. Moreover, most miRNA studies pertaining to age-related changes lack robust causal evidence, necessitating parallel in vivo studies utilizing gain- or loss-of-function animal models to establish causal links between associated molecular signatures and the aging process. While efforts in model organisms such as flies and nematodes have been showcased^4^, comprehensive evaluations in mouse models are needed. Notably, our group demonstrated lethality and neural tube patterning abnormalities in *Mir-23-27-24* double knockout mice^27,29^, demonstrating the critical roles of these miRNAs in embryos. Given that *Mir-23-27-24* floxed mice have also been generated^104–107^, they represent an ideal platform for further exploration of the tissue-specific functions of the *Mir-23-27-24* clusters in adult mice and their relevance to tissue aging. Furthermore, given the distinct roles of individual miRNAs within the *Mir-23-27-24* cluster, dissecting their collective and individual contributions to aging through the generation of individual miRNA knockout mice represents an intriguing avenue for cooperative investigation. Second, an important question arises regarding the aging “regulome” governed by the *Mir-23-27-24* cluster, i.e., is there a tissue-specific miRNA-mediated aging regulome or is there a common aging regulome across different tissues? Advances in single-cell multiomics techniques hold promise for resolving this issue in the near future. Finally, the potential of miRNA-based therapies in ameliorating neurodegenerative diseases or intervening in the aging process warrants consideration, especially in light of the prominence of RNA vaccines post-COVID-19. This topic is at the forefront of pharmaceutical focus in the coming years.
